# High-Throughput Screening to Identify Novel Compounds Affecting the Genome Editing Efficiency of CRISPR System

**DOI:** 10.3390/molecules30081811

**Published:** 2025-04-17

**Authors:** Jiasong Chang, Xiulong Yang, Tong Zhang, Hao Sun, Hongying Cheng, Zhangrong Jia, Yiying Li, Sanyuan Ma, Teng Sun, Jimin Cao

**Affiliations:** 1 Key Laboratory of Cellular Physiology, Shanxi Medical University, Ministry of Education, Taiyuan 030001, China; jiasongchang@163.com (J.C.); yxl1551006602@163.com (X.Y.); jiazhangrong@163.com (Z.J.); yyingli@aliyun.com (Y.L.); 2 Department of Physiology, Shanxi Medical University, Taiyuan 030001, China; 3 Biological Science Research Center, Southwest University, Chongqing 400715, China; zt137703197@email.swu.edu.cn (T.Z.); sunhao19971204@163.com (H.S.); masy@swu.edu.cn (S.M.); 4 Department of Preschool Education, Lvliang Teachers College, Lvliang 033001, China; chenghy_2025@163.com

**Keywords:** high-throughput screening, CRISPR/Cas9, off-target effect, single-strand annealing, compound

## Abstract

Genome editing is a promising therapeutic strategy for genetic disorders by modifying the genome precisely, especially the CRISPR/Cas9 system. However, a major limitation of CRISPR/Cas9 in gene therapy is the biosafety issues caused by off-target effects. Compounds that can modulate the genome editing efficiency of the CRISPR/Cas9 system, especially those reducing the off-target effects, are potentially useful pharmacological tools for improving the effectiveness and safety of genome editing. Here, we performed high-throughput screening in HEK 293FT cells to discover compounds that decrease or increase the genome editing efficiency of the CRISPR/Cas9 system from 9930 compounds. After two rounds of screening, we identified that CP-724714, a ErbB2 (HER2) tyrosine kinase inhibitor, decreased the CRISPR/Cas9 efficiency and reduced the off-target effects by suppressing the efficiency of CRISPR/Cas9, and was thus named a CRISPR decelerator (or inhibitor), while Clofarabine, a DNA synthesis inhibitor, increased the efficiency of CRISPR/Cas9, and was named a CRISPR accelerator. We further identified four compounds (Tranilast, Cerulenin, Rosolic acid and Resveratrol) that affected the efficiency of single-strand annealing (SSA) repair. Among them, Tranilast, Cerulenin and Rosolic acid are potential SSA decelerators, while Resveratrol is a potential SSA accelerator. These identified compounds may be useful in optimizing mammalian genetic manipulation techniques.

## 1. Introduction

Genome variations can cause a variety of genetic diseases [1,2,3]. There are thousands of known human genetic diseases; most of them are single-gene mutations, while some others are multiple-gene mutations. Currently, there are few effective treatments for most genetic diseases. Traditional methods for treating genetic diseases are mainly the applications of small molecules or enzyme replacement therapies. These therapies can only alleviate the clinical manifestations but cannot cure the diseases [4]. Because the root cause of genetic diseases is gene mutation, the most powerful therapeutic strategy for genetic diseases is gene therapy [5]. Early useful gene therapy involved replacing the endogenous defective DNA with exogenous “correct” DNA [6,7]. However, there are several serious defects that limit the application of gene therapy, such as the difficulty of pouring large foreign genes into cells and the potential risks of foreign genes [8,9]. An alternative means of gene therapy is the knockdown of defective genes by RNA interference (RNAi) [10]. However, gene knockdown still has limitations such as off-target effects and an inability to modify the genome, which would obstruct the application of RNAi in the treatment of genetic diseases.

In the past decade, genome editing technologies have developed rapidly and have offered hopefulness in treating genetic diseases [11,12,13]. In particular, the clustered, regularly interspaced short palindromic repeats/clustered, regularly interspaced short palindromic repeat-associated 9 (CRISPR/Cas9) system has brought unprecedented light to gene therapy because of its easily programmable character. The CRISPR/Cas system is an adaptive immune system discovered in bacteria and archaea [14,15], and it has been developed as an effective programmed genome editing tool in eukaryotes [16,17,18,19,20,21,22,23,24]. In recent years, the CRISPR/Cas9 system has been confirmed to be an efficient tool in gene therapies for Parkinson’s disease (PD) [25,26,27], hereditary angioedema [13,28], hereditary tyrosinaemia [29], progressive hearing loss [30], hemophilia A [31,32], β-thalassemia [33,34], α1-antitrypsin [35] and Duchenne muscular dystrophy (DMD) [36,37]. To improve the efficiency of gene targeting by CRISPR/Cas9, several strategies have been tried. Weina Zhang et al. performed a high-throughput small-molecule screening that identifies as a potentiator of CRISPR/Cas9-mediated genome editing [38]. However, multiple studies have pointed out that the CRISPR/Cas9 system has significant off-target effects [39,40,41,42], a critical defect of gene therapy. To overcome this drawback, several strategies have been tried. In some cases, improving the specificity of the sgRNA sequence design [43,44] could avoid the off-target effects. The other strategy for improving genome editing specificity is to produce two single-strand breaks on the DNA strands using Cas9n [45,46] or dCas9-*Fok* I [47,48]. In addition, shortening the working time of CRISPR/Cas9 by inhibiting its activity could be an alternative effective approach to reduce potential off-target effects. There are several strategies to inhibit the CRISPR system, such as the applications of anti-CRISPR proteins identified from bacteria [49,50,51] and bacteriophages [52], small-molecule compound inhibitors of CRISPR/Cas9 [53], oligonucleotide inhibitors of CRISPR-Cpf1 [54], a light-inducible CRISPR/Cas9 system [55,56], synthetic amino acid-dependent Cas9 transcription [57] and linking N-terminal geminin to shorten the half-life of Cas9 [58]. Among these methods, small compounds may have better prospects in manipulating the activity of CRISPR because of their advantages in designing, production, time-saving and cost.

Here, we constructed a high-throughput platform, and using this platform, we screened 9930 compounds and finally identified two compounds (CP-724714 and Clofarabine) that could effectively affect the genome editing efficiency of the CRISPR/Cas9 system, with CP-724714 decelerating and Clofarabine accelerating the CRISPR/Cas9 efficiency. We also identified four compounds (Tranilast, Cerulenin, Rosolic acid and Resveratrol) that could effectively affect the SSA repair efficiency. These compounds may have the potential to be developed as pharmacological tools in manipulating the genome editing efficiency of the CRISPR/Cas9 system in the future.

## 2. Results

### 2.1. High-Throughput Platform for Screening Compounds Modulating CRISPR Efficiency

**Figure 1:**
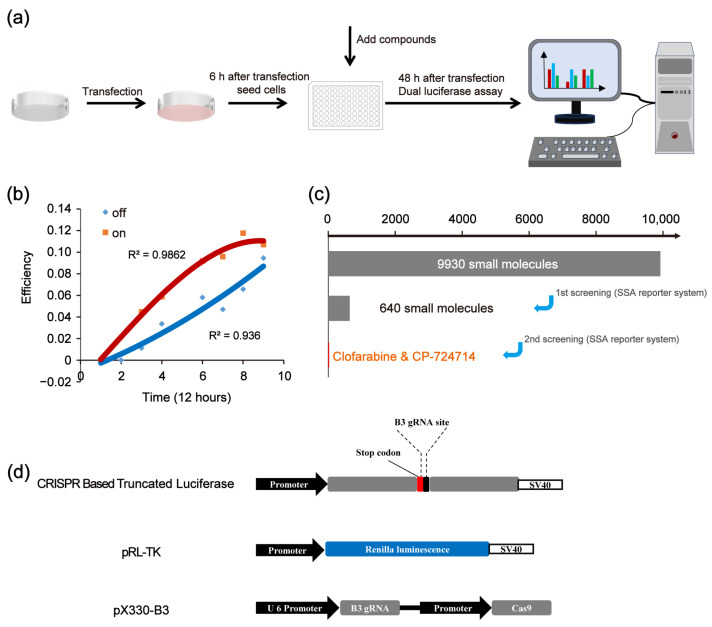
Overview of the screening strategy. (**a**) Scheme of experimental procedures. (**b**) Effects of CRISPR working times on the off-target effects. (**c**) Scheme of in vitro screening to identify CRISPR/Cas9 modulators. (**d**) Vectors of CRISPR-based SSA.

We first established a high-throughput platform for screening compounds that affect the CRISPR/Cas9 system. The platform was constructed based on the single-strand annealing (SSA) reporter activation via the CRISPR/Cas9 system. Then, we used the platform to edit the genes of the HEK 293FT cells. A scheme of the experimental procedure is shown in Figure 1a. The initial results show that, along with the prolongation of the CRISPR/Cas9 working time, the increasing trend of on-target efficiency was slowed down, while the increasing trend of off-target efficiency became more pronounced (Figure 1b). This phenomenon may be attributed to the fact that CRISPR/Cas9 preferentially targets specific sites. Once the target site has been edited, the CRISPR/Cas9 system is unable to effectively bind to the target site, leading to an increase in the off-target effects. Therefore, appropriately shortening the working time of CRISPR may reduce the off-target effects and maintain the on-target efficiency. Figure 1c shows the scheme of the in vitro screening to identify CRISPR/Cas9 modulators.

**Figure 2:**
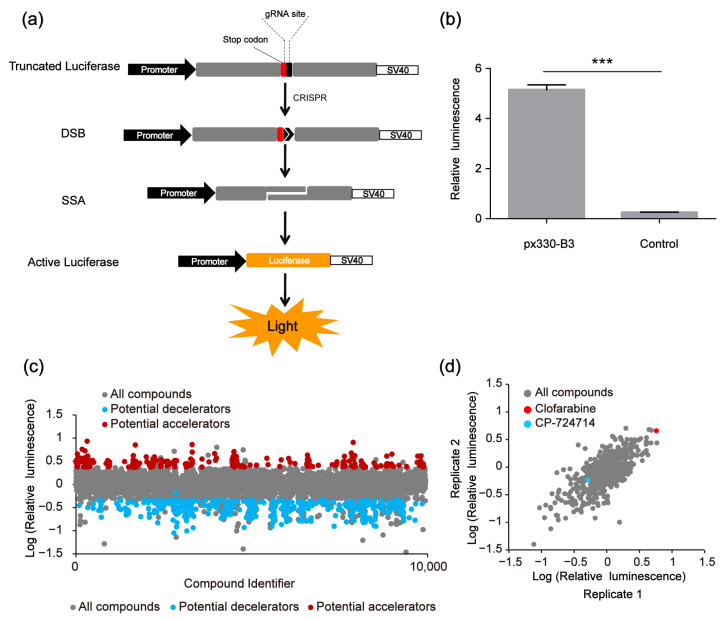
High-throughput screening to identify compound modulators of CRISPR/Cas9 efficiency based on the single-strand annealing (SSA) assay. (**a**) Scheme of CRISPR-based SSA reporter system combined with dual luciferase (firefly luciferase and Renilla luciferase). (**b**) Relative luminescence of CRISPR-based SSA. HEK 293FT cells were divided into two groups, the experimental group was transfected with the CRISPR-based SSA reporter vector and pX330-B3, the control group was transfected with the SSA reporter vector alone. Forty-eight hours after transfection, the dual luciferase (firefly luciferase and Renilla luciferase) activities were detected by the Promega GloMax-Multi Instrument. The statistical significant differences were determined by Student’s *t*-test (*** *p* < 0.001). (**c**) The first-round screening to identify CRISPR/Cas9 modulators from 9930 compounds. (**d**) The second-round screening of the 640 compounds.

The used SSA reporter system consisted of three parts: the SSA reporter vector, the Renilla luciferase expression vector pRL-TK and the all-in-one CRISPR vector pX330 (containing *Sp*Cas9 protein expression cassette and sgRNA expression cassette) (Figure 1d). The SSA reporter vector, pRL-TK and pX330 were co-transfected into HEK 293FT cells. Six hours later, the transfected cells were seeded into 96-well black plates (about 3000 cells/well), with each well containing a known compound (10 μM). The CRISPR system would cleave the DNA double helix of the SSA reporter vector at the target site and activate the double-strand DNA break (DSB) repair pathway. Since the two flanks of the DSBs were overlapped in about 871 bp, the DSBs would be repaired by SSA and the firefly luciferase gene was repaired. Forty-eight hours after transfection, the dual luciferase (firefly luciferase and Renilla luciferase) activity was detected by the Promega GloMax-Multi Instrument. In our platform, the genome editing efficiency of CRISPR/Cas9 was calculated based on the luciferase activity (Figure 2a). After several repeated experiments and preliminary verification, we chose the highly specific sgRNA, i.e., the B3 target site, as the target site of the SSA reporter system to perform the subsequent screening (Figure 2b).

### 2.2. Potential CRISPR Decelerators and Accelerators Identified by Two-Rounds Screening

**Figure 3:**
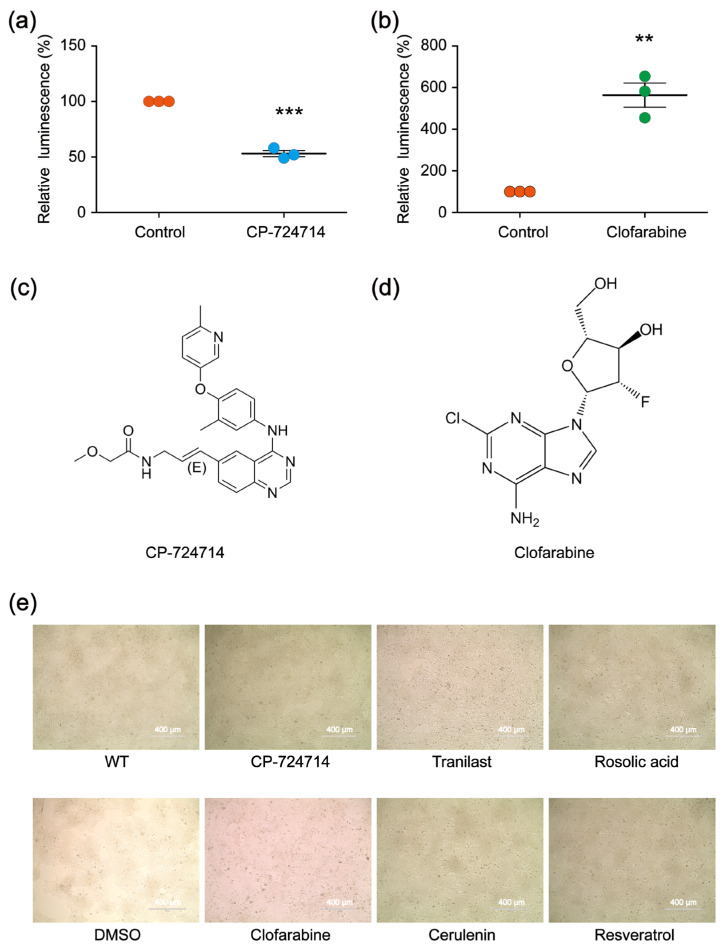
(**a**,**b**) The modulatory effects of CP-724714 and Clofarabine on the CRISPR/Cas9 efficiency, validated by relative luminescence reporter assays. The statistical significant differences were determined by Student’s *t*-test (** *p* < 0.01, *** *p* < 0.001). (**c**,**d**) Chemical structures of CP-724714 and Clofarabine. (**e**) Microscopic evaluation showing that the cytotoxic effects of the compounds were very low in HEK 293FT cells.

In the first-round screening, 9930 small molecules were detected through our platform, and 640 compounds were identified as the potential candidates that might affect the genome editing efficiency of CRISPR/Cas9. Among them, 400 small molecules were potential decelerators (inhibitors) of the CRISPR system, while 240 small molecules were potential accelerators of the CRISPR system (Figure 2c). Afterwards, the 640 compounds underwent second-round screening through the same platform to test the effect of the small molecules on the genome editing efficiency (Figure 2d). Finally, two compounds (CP-724714 and Clofarabine) were selected that might have higher potentialities, respectively, as a decelerator or an accelerator of the CRISPR/Cas9 system (Figure 3a–d).

To further test the effect of CP-724714 on the genome editing efficiency, we constructed a CRISPR vector (pX330-FANCF) that could target the endogenous site of the HEK 293FT cells. The vector was transfected into the HEK 293FT cells. Six hours after transfection, the transfected HEK 293FT cells were evenly seeded into two wells, with one well containing CP-724714 as the experimental group and the other well containing Dimethyl sulfoxide (DMSO) as the control group. Forty-eight hours later, all the cells were collected to extract genomic DNA. Then, the targeting region of FANCF was amplified by polymerase chain reaction (PCR), and the effects of the CP-724714 on the genome editing efficiency were detected using next-generation sequencing (NGS). By analyzing the NGS data, we found that CP-724714 could reduce the CRISPR efficiency to 93.0% (Table 1). We also constructed an alternative CRISPR vector (pX330-VEGFA) that could target the endogenous site of the HEK 293FT cells, aiming to test the effect of Clofarabine on the genome editing efficiency using the same experimental protocol. By analyzing the NGS data, we found that Clofarabine could increase CRISPR efficiency to 214.4% (Table 2). Microscopic analysis showed that the two compounds exhibited little cytotoxicity in the HEK 293FT cells (Figure 3e).

### 2.3. Compounds Affecting the SSA Efficiency

**Figure 4:**
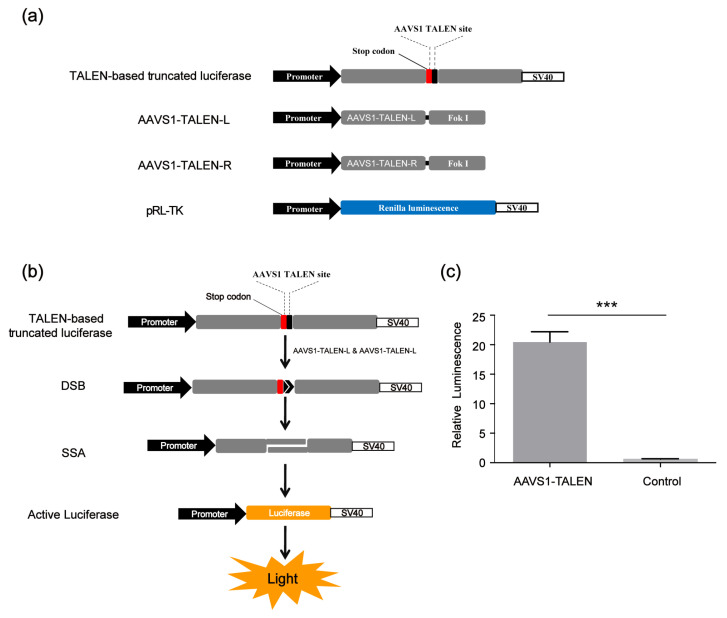
TALEN-based SSA reporter system. (**a**) Vectors of TALEN-based SSA. (**b**) Scheme of CRISPR-based SSA reporter system combined with dual luciferase (firefly luciferase and Renilla luciferase). (**c**) Relative luminescence of TALEN-based SSA. HEK 293FT cells were divided into two groups: the experimental group was transfected with TALEN-based SSA reporter vector and AAVS1- TALEN vector; the control group was transfected with TALEN-based SSA reporter vector alone. Forty-eight hours after transfection, the dual luciferase (firefly luciferase and Renilla luciferase) activities were detected by the Promega (GloMax-Multi Instrument). The statistical significant differences were determined by Student’s *t*-test (*** *p* < 0.001).

Since our screening platform was based on the SSA assay, in addition to screening CRISPR/Cas9 modulators, we also identified compounds that could affect the SSA repair efficiency. To improve the screening reliability of the SSA decelerator/accelerator and exclude those compounds affecting the CRISPR/Cas9 system, we developed a TALEN-based SSA reporter system consisting of four vectors, the SSA reporter vector (TALEN-based truncated luciferase), the Renilla luciferase expression vector pRL-TK and two AAVS1-TALEN vectors (AAVS1-TALEN-L and AAVS1-TALEN-R) (Figure 4a). This system could cleave the DNA double helix of the SSA reporter vector at the target site and activate the single-strand annealing (SSA); then, the firefly luciferase gene was repaired as shown in Figure 4b. The TALEN-based SSA reporter system worked well (Figure 4c).

**Figure 5:**
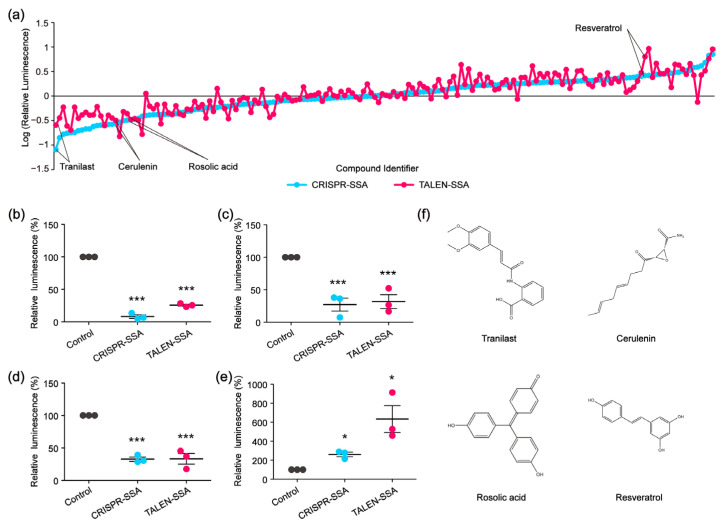
High-throughput screening to identify SSA decelerators and accelerators. (**a**) Line chart showing the SSA decelerators and accelerators identified by CRISPR or TALEN-based SSA reporter system. (**b**–**e**) The modulatory effects of Tranilast (**b**), Cerulenin (**c**), Rosolic acid (**d**) and Resveratrol (**e**) on the SSA efficiency validated by relative luminescence reporter assays (statistical significance was assessed using Student’s *t*-test. * *p* < 0.05; *** *p* < 0.001). (**f**) Chemical structures of Tranilast, Cerulenin, Rosolic acid and Resveratrol.

To identify compounds that could decelerate or accelerate the SSA repair efficiency, we re-screened 159 compounds that affected the CRISPR-based SSA using the TALEN-based SSA reporter system. Among the 159 compounds, 78 compounds decelerated the CRISPR-based SSA, while 81 compounds accelerated the CRISPR-based SSA. As we expected, not all the CRISPR-based SSA modulators were decelerators of the TALEN-based SSA. Similarly, some CRISPR-based SSA accelerators could not enhance the TALEN-based SSA (Figure 5a). We then selected several compounds that could simultaneously accelerate both the CRISPR-based SSA and TALEN-based SSA, as well as several compounds that could decelerate both the CRISPR-based SSA and the TALEN-based SSA at the same time. Of these compounds, Tranilast, Cerulenin and Rosolic acid could potentially decelerate the SSA efficiency, whereas Resveratrol could distinctly accelerate the SSA efficiency (Figure 5b–f). Tranilast is a tryptophan metabolite analog and is commonly used as an antihistamine drug. Cerulenin is an inhibitor of natural fatty acid synthase (FASN) and can inhibit the catalytic activity of topoisomerase I and enhance SN-38-induced apoptosis. Rosolic acid is an acid-base indicator and has been used to induce cellular stress in HEK293 cells. Resveratrol is a natural polyphenol with an antioxidant effect. Here, we further found that these four compounds are potential modulators of SSA efficiency. However, the mechanisms by which these compounds affect SSA efficiency are still unclear and warrant further investigation. In addition, microscopic analysis shows that these four compounds were not cytotoxic in HEK 293FT cells, as shown in Figure 3e.

## 3. Discussion

Genetic diseases pose a serious threat to human health and so far, few effective therapies are available. Gene therapy is considered the most effective therapeutic means of treating genetic diseases. In the last decade, genome editing tools have been developing rapidly, which could precisely modify the genome DNA. As the most simple and programmable site-specific gene engineering system, CRISPR/Cas9 has the potential to effectively cure genetic diseases [59]. However, the CRISPR/Cas9 system has raised biosafety concerns because of its off-target effects, which may limit its use in gene therapy.

To reduce the off-target effects of the CRISPR system, researchers have made various attempts. Because abnormal sgRNA binding can lead to off-target effects, researchers have improved the specificity of the sgRNA sequence [60,61]. Additionally, producing two single-strand breaks on different DNA strands using Cas9n or dCas9-*Fok* 1 can also reduce the off-target effects. Alternatively, appropriate shortening of the working time of the CRISPR/Cas9 system may also reduce the off-target effects. Several strategies have been used to limit the working time of CRISPR, such as the utilizations of anti-CRISPR proteins [62], conditional Cas9 variants and linking N-terminal Geminin to shorten the half-life of Cas9 [63], and compound inhibitors of CRISPR/Cas9 [53]. Compound inhibitors hold great promise in reducing the off-target effects of the CRISPR system due to their ease of administration and low cost. The present study aimed to discover new compound modulators of CRISPR/Cas9 activity, including inhibitors/decelerators and accelerators.

Using the self-constructed high-throughput platform, we screened out some novel compounds that could affect the genome editing efficiency of CRISPR/Cas9. After two rounds of screening via the SSA reporter system, we identified two potential compounds (CP-724714 and Clofarabine) that are likely a decelerator and an accelerator of CRISPR/Cas9, respectively. CP-724714 could inhibit the efficiency of CRISPR, and Clofarabine could increase the efficiency of CRISPR. CP-724714 is a ErbB2 (HER2) tyrosine kinase inhibitor [64]; here, we first identified that it is also an decelerator of CRISPR efficiency. Clofarabine is originally known as a DNA synthesis inhibitor [65], and we found for the first time that it is a potential accelerator of CRISPR efficiency. In addition, we identified three potential SSA decelerators (Tranilast, Cerulenin and Rosolic acid) and one potential SSA accelerator (Resveratrol), which might be useful for manipulating the SSA efficiency in genome editing.

We attempted to explain the relationship between the two novel compounds (CP-724714 and Clofarabine) and DNA damage repair. CP-724714 is an ErbB2 (HER2) tyrosine kinase inhibitor; here, we first identified that it is also an decelerator of CRISPR efficiency. Little research has been conducted on CP-724714 in relation to DNA damage repair. Clofarabine is originally known as a DNA synthesis inhibitor. The DNA synthesis and repair could be inhibited by Clofarabine via the inhibition of ribonucleotide reductase and DNA polymerase [66]. In recent years, Clofarabine has been used in research for the treatment of refractory acute leukemias [67]. The genome editing via CRISPR requires Cas9 binding, cutting and the appropriate DNA damage repair pathway. We hypothesize that Clofarabine may enhance the genome editing efficiency of CRISPR by disrupting the DNA damage repair pathway.

This study has several limitations. We only tested the modulatory effects of the above compounds on the genome editing efficiency of CRISPR/Cas9 at a cellular level. Validations of these effects on the integrative level and even clinical level are warranted. The molecular mechanisms by which these compounds exert their modulatory effects on CRISPR/Cas9 efficiency were also not investigated and need further study. The reporter used in this study requires Cas9 binding, cutting and the appropriate repair pathway. Modification of the repair pathways is the most likely mechanism by which these compounds exert their modulatory effects on CRISPR/Cas9 efficiency. Since DNA repair pathways vary widely between cell types, whether these compounds exert their modulatory effects on CRISPR/Cas9 efficiency in other cell types also needs further investigate.

In summary, we reported a high-throughput screening platform, and using this platform and HEK 293FT cells, we identified two compound modulators of CRISPR/Cas9 efficiency, i.e., CP-724714 and Clofarabine. CP-724714 decelerated, whereas Clofarabine accelerated, the genome editing efficiency of CRISPR/Cas9. We also identified four compounds that could modulate the SSA repair efficiency. Among the four compounds, Tranilast, Cerulenin and Rosolic acid are potential SSA decelerators, while Resveratrol is a potential SSA accelerator. These six compounds may have prospects to be developed as small molecule tools for manipulating the genome editing efficiency of the CRISPR/Cas9 system and reducing its off-target effects.

## 4. Materials and Methods

### 4.1. Design and Construction of Vectors

The SSA reporter vector (T-CMV-SSA-luciferase) was constructed based on the firefly luciferase derived by the cytomegalovirus (CMV) promoter. The firefly luciferase coding sequence was divided into two parts based on direct repeats as SSA arms: the first part contained 1188 bp with a stop codon at the end of the sequence and the second part contained 1336 bp. In addition, an 871 bp sequence of firefly luciferase was overlapped between the two parts of the firefly luciferase coding sequence, and a sgRNA target site was inserted into the direct repeats. The CMV promoter was amplified with primers (the F primer was CMV-SSA-1F and the R primer was CMV-SSA-1R) from lentiCRISPR v2 (Addgene, Watertown, MA, USA, cat. no. 52961). The first part of the firefly luciferase coding sequences was amplified with primers [CMV-SSA-2F and B1R(A4-SSA-2R)] from pGL3-Enhancer (E1771, Promega, Madison, WI, USA). The space sequences were amplified with primers (B2F and B2R) from *pEASY*^®^-T5 Zero (CT501-01, Transgene, Shenzhen, China). These three fragments were linked together using overlap PCR with primers (CMV-SSA-1F and B3R) and inserted into *pEASY*^®^-T5 Zero, named vector-21. The second part of the firefly luciferase coding sequences was amplified with primers [B4F(A4-SSA-3F) and PM-R] from pGL3-Enhancer (E1771, Promega, Madison, WI, USA) and inserted into *pEASY*^®^-T5 Zero, named vector-22. Subsequently, vector-22 was cloned into the *Hind* III and *Nhe* I sites of vector-21 to generate the SSA reporter vector, named T-CMV-SSA-luciferase. The Renilla luciferase expression vector pRL-TK (E2241) was purchased from Promega (Madison, WI, USA). The CRISPR vector pX330-U6-Chimeric_BB-CBh-hSpCas9 was provided by Addgene (Plasmid #42230) [17]. To construct the CRISPR vector, pX330-FANCF, pX330-VEGFA and pX330-B3, all the three sgRNA sequences, FANCF, VEGFA and B3, were synthesized as oligomers, then annealed and inserted into the *Bbs* I site of pX330-U6-Chimeric_BB-CBh-hSpCas9. The B3 and AAVS1 target sites were synthesized as oligomers, then annealed and inserted into the *Age* I and *Hind* III sites of T-CMV-SSA-luciferase to form T-CMV-SSA-luciferase-B3 and T-CMV-SSA-luciferase-AAVS1. The TALEN expression vectors, AAVS1-TALEN-L (#59025) and AAVS1-TALEN-R (#59026), were provided by Addgene [68]. The primers used for constructing the vectors are listed in Tables S3–S5.

### 4.2. Cell Culture and Transfection

The HEK 293FT cell line stored in our laboratory was cultured with DMEM (Dulbecco’s modified Eagle’s medium) plus GlutaMAX medium (ThermoFisher Scientific, Billerica, MA, USA) containing 10% (*v*/*v*) FBS (fetal bovine serum) at 37 °C in a 5% (*v*/*v*) CO_2_ atmosphere. The culture media were changed every day. HEK 293FT cells were seeded into well plates (Corning, Corning, NY, USA) and transfected with plasmids using Lipofectamine 2000 (ThermoFisher Scientific) according to the manufacturer’s instructions.

### 4.3. Dual Luciferase Reporter Assay

HEK 293FT cells were transfected with dual luciferase reporter vectors using Lipofectamine 2000. Six hours later, the cells were seeded into 96-well black plates, and each well contained a known compound. Forty-six hours later, the firefly luciferase and Renilla luciferase activity in each well was detected using the Dual-Glo luciferase assay system (Promega, Madison, WI, USA) with a Promega GloMax-Multi Instrument following the manufacturer’s instructions.

### 4.4. Next-Generation Sequencing

To perform the next-generation sequencing, all the groups (control groups and compound groups for the target sites) of the HEK 293FT cells were, respectively, collected, and all the genomic DNA was extracted. Afterwards, the genomic DNA of all the groups was PCR-amplified using PrimeSTAR^®^ Max DNA Polymerase (Takara, Kusatsu City, Shiga Prefecture, Japan) with primer matrix for NGS (next-generation sequencing) (Table S4). All the PCR products were gel-purified for the NGS using the paired-end 150 bp strategy (Illumina strategy, Mega Genomic services, Beijing, China). The sequencing depth was set at more than 10,000× to ensure accurate variant detection. After the next-generation sequencing, all the raw data were uploaded to the server; then, they were filtered using the Trimmomatic-0.35.jar trimming software and the paired sequences were spliced by a flash tool. Finally, the efficiency of CRISPR was calculated, respectively.

### 4.5. Compounds Libraries

The small compound libraries used in this study were obtained from the National Compound Resource Center (Shanghai, China). These libraries included the Kinase Inhibitor Library, Syn Kinase Inhibitors, Phosphatase Inhibitor Library, Protease Inhibitor Library, Nuclear Receptor Ligand Agonists or Antagonists Library, SIGMA LOPAC Natural Products Library, NCC-001_Shipment, ICCB Known Bioactives Library, Orphan Ligand Library, REDOX Library, ynxl2080-ncds, FDA-Approved Drug Library, Epigenetics Library, Protein Kinase Inhibitor Library, Stem Cell Regulators Library, IBscreening library, Prestwick Chemical Library, TocriscreenTotal and The Spectrum Collection. In total, 9930 small molecules were screened in this study. The compound identification numbers (IDs) are shown in Table S5a, and the library names of the compounds are shown in Table S5b.

### 4.6. The Efficiency of on/off Target

We constructed a vector PX330-V1 for knocking out the on-target SSA reporter vector (contains the target site “GGGTGGGGGGAGTTTGCTCCTGG”) and the off-target SSA reporter vector (contains the off-target site with mismatched bases “GGGAGGGTGGAGTTTGCTCCTGG”) in the HEK 293FT cells. After transfecting the HEK 293FT cells, cells were collected every 12 h. The efficiency of the on-target or off-target effects of CRISPR/Cas9 was detected by the Dual luciferase reporter assay (relative luminescence).

## 5. Conclusions

In this work, we constructed a high-throughput platform for screening compounds modulating CRISPR efficiency. We identified two novel compound modulators of CRISPR/Cas9 efficiency. CP-724714 decreased the CRISPR/Cas9 efficiency, while Clofarabine increased the efficiency of CRISPR/Cas9. We further identified four novel compounds (Tranilast, Cerulenin, Rosolic acid and Resveratrol) that could affect the efficiency of SSA repair. The compounds identified in this work can serve as small molecule tools to manipulate the genome editing efficiency of the CRISPR/Cas9 system and reduce off-target effects.

## Figures and Tables

**Table 1:** Deep sequencing to identify CRISPR/Cas9 decelerators.

Target Site	WT	FANCF	FANCF/WT
CP-724714	9.12%	8.48%	93.03%

**Table 2:** Deep sequencing to identify CRISPR/Cas9 accelerators.

Target Site	WT	VEGFA	VEGFA/WT
Clofarabine	5.85%	2.73%	214.41%

## Data Availability

The datasets used in the study are available from the corresponding author on reasonable request. The raw data generated in this study have been submitted to the NCBI BioProject database under accession number PRJNA1209650.

## Supplementary Materials

The following supporting information can be downloaded at: https://www.mdpi.com/article/10.3390/molecules30081811/s1, Table S1: Sequences of target sites for CRISPR; Table S2: Sequences of target sites for TALEN; Table S3: Sequences of primers used for vectors; Table S4: Sequences of primers used for next-generation sequencing; Table S5: The IDs and relative luminescence activities (log value) of the 9930 compounds.
