# Supplementary material for: High-Throughput Screening to Identify Novel Compounds Affecting the Genome Editing Efficiency of CRISPR System
Source: Molecules. 2025 Apr 17;30(8):1811. doi: 10.3390/molecules30081811 (PMC12029788; doi:10.3390/molecules30081811)
Supplement: Supplementary file 1 [file molecules-30-01811-s001.zip › Supplementary Material.pdf]

Supplementary Material

High-throughput screening to identify novel compounds affecting the genome editing efficiency of CRISPR system

Jia-Song Chang[1,2,‡], Xiu-Long Yang[1,2,‡], Tong Zhang[3], Hao Sun[3], Hong-Ying Cheng[4], Zhang-Rong Jia[1,2], Yi-Ying Li[1,2], Teng Sun[1,2*], San-Yuan Ma[3,*], Ji-Min Cao[1,2,*]

[1] Key Laboratory of Cellular Physiology at Shanxi Medical University, Ministry of Education, Taiyuan, China

[2] Department of Physiology, Shanxi Medical University, Taiyuan, China

[3] Biological Science Research Center, Southwest University, Chongqing, China

[4] Department of Art, Lvliang Normal University, Lvliang, China

‡ These authors contributed equally to this work.

* Corresponding authors.

E-mail address: caojimin@sxmu.edu.cn (J.C., lead contact); masy@swu.edu.cn (S.M.); tengsun@sxmu.edu.cn (T.S.)

Supplementary Tables

Table S1 Sequences of target sites for CRISPR

Target sites name	Target site
B3	GGAACACTACATGCTGCTTGAGG
FANCF	GGAATCCCTTCTGCAGCACCTGG
VEGFA	GACCCCCTCCACCCCGCCTCCGG
V1 on target	GGGTGGGGGGAGTTTGCTCCTGG
V1 off target	GGGAGGGTGGAGTTTGCTCCTGG

Table S2 Sequences of target sites for TALEN

Target sites name	Target site
TALEN-AAVS1	GGGGCTTTTCTGTCACCAATCCTGTCCCTAGTGGCCCCACTGTGGGGTGGAGGGGACAGAT
AAVS1-TALEN-L targeting	CCCCTCCACCCCACAGT
AAVS1-TALEN-R targeting	TTTCTGTCACCAATCCT

Table S3 Sequences of primers used for vectors

Primer name	Primer sequence
CMV-SSA-1F	5-GTCGACCCGCTCGAGACGCGTT
CMV-SSA-1R	5-CTTTATGTTTTTGGCGTCTTCCATTACGAAGACTACTAGGAGCTCTG
CMV-SSA-2F	5-CAGAGCTCCTAGTAGTCTTCGTAATGGAAGACGCCAAAAACATAAAG
B1R(A4-SSA-2R)	5-AGATCTAGGGCCCTACCGGTTCACATAGGACCTCTCACACACAGTT
B2F	5-TGAACCGGTAGGGCCCTAGATCTTCTTGACGAGTTCTTCTGAATTAT
B2R	5-GCTAGCAAAGCTTTCCCGGGAGGTACCGCATTGGTAACTGTCAGACCA
B3R	5-GCTAGCAAAGCTTTCCCG
B4F(A4-SSA-3F)	5-AAGCTTTCGACATTTATAATGAACGTGAAT
PM-R	5-ATGCTAGCTACCACATTTGTAGAGGTTTTACTT
SSA-AAVS1-1F	5-CCGGGGGGCTTTTCTGTCACCAATCCTGTCCCTAGTGGCCCCACTGTGGGGTGGAGGGGACAGAT
SSA-AAVS1-1R	5-AGCTATCTGTCCCCTCCACCCCACAGTGGGGCCACTAGGGACAGGATTGGTGACAGAAAAGCCCC
SSA-B3F	5-CCGGGGAACACTACATGCTGCTTGAGG
SSA-B3R	5-AGCTCCTCAAGCAGCATGTAGTGTTCC

Table S4 Sequences of primers used for next generation sequencing

Group name	Primer name	Primer sequence
CP-724714	VV-F5	5-GGTTTGCACATGCAGCCCCAGCTACCACC
CP-724714	VV-R7	5-AGTTCATACGGCTCCTCCGAAGCGAGAACA
Control group	VV-F8	5-CAGGAACCAGGACAGCCCCAGCTACCACC
Control group	VV-F9	5-TGCTCGATGTGCCAGCCCCAGCTACCACC
Control group	VV-R8	5-GGTGAGCAAGCATCCTCCGAAGCGAGAACA
Control group	VV-R9	5-AGTGACTGTCAATCCTCCGAAGCGAGAACA
Clofarabine	F-F6	5-TGGGTTAACACATCCCAGGTGCTGACGTAG
Clofarabine	F-R2	5-GCTACAAGCCCTGATGTTCCAATCAGTACGCAG
Control group	F-F8	5-CAGGAACCAGGATCCCAGGTGCTGACGTAG
Control group	F-F9	5-TGCTCGATGTGCTCCCAGGTGCTGACGTAG
Control group	F-R8	5-GGTGAGCAAGCAGATGTTCCAATCAGTACGCAG
Control group	F-R9	5-AGTGACTGTCAAGATGTTCCAATCAGTACGCAG

Table S5 The IDs and relative luminescence activities (log value) of the 9,930 compounds (This is large Excel file including sheet a and sheet b and shows separately)

Supplementary Sequences

> T-CMV-SSA-luciferase

Green: CMV promoter. Gray: The first SSA arm. Blue: The second SSA arm. Yellow: SV40PA.

Note: sequences shown by red fonts indicate the *Age* I and *Hind* III cutting sites.

GTCGAC<u>CCGCTCGAGACGCGTT</u>GACATTGATTATTGACTAGTTATTAATAGTAATCAATTACGGGGTCATTAGTTCATAGCCCATATATGGAGTTCCGCGTTACATAACTTACGGTAAATGGCCCGCCTGGCTGACCGCCCAACGACCCCCGCCCATTGACGTCAA

TAATGACGTATGTTCCCATAGTAACGCCAATAGGGACTTTCCATTGACGTCAATGGGTGGACTATTTACGGTAAACTGCCCACTTGGCAGTACATCAAGTGTATCATATGCCAAGTACGCCCCCTATTGACGTCAATGACGGTAAATGGCCCGCCTGGCATTATGC

CCAGTACATGACCTTATGGGACTTTCCTACTTGGCAGTACATCTACGTATTAGTCATCGCTATTACCATGGTGATGCGGTTTTGGCAGTACATCAATGGGCGTGGATAGCGGTTTGACTCACGGGGATTTCCAAGTCTCCACCCCATTGACGTCAATGGGAGTTTGT

TTTGGCACCAAAATCAACGGGACTTTCCAAAATGTCGTAACAACTCCGCCCCATTGACGCAAATGGGCGGTAGGCGTGTACGGTGGGAGGTCTATATAAG<u>CAGAGCTCCTAGTAGTCTTCGTA</u>ATG<u>GAAGACGCCAAAAACATAAAG</u>AAAGGCCCGGCGCCATT

CTATCCGCTGGAAGATGGAACCGCTGGAGAGCAACTGCATAAGGCTATGAAGAGATACGCCCTGGTTCCTGGAACAATTGCTTTTACAGATGCACATATCGAGGTGGACATCACTTACGCTGAGTACTTCGAAATGTCCGTTCGGTTGGCAGAAGCTATGAAACG

ATATGGGCTGAATACAAATCACAGAATCGTCGTATGCAGTGAAAACTCTCTTCAATTCTTTATGCCGGTGTTGGGCGCGTTATTTATCGGAGTTGCAGTTGCGCCCGCGAA<u>CGACATTTATAATGAACGTGAAT</u>TGCTCAACAGTATGGGCATTTCGCAGCCTACC

GTGGTGTTCGTTTCCAAAAAGGGGTTGCAAAAAATTTTGAACGTGCAAAAAAAGCTCCCAATCATCCAAAAAATTATTATCATGGATTCTAAAACGGATTACCAGGGATTTCAGTCGATGTACACGTTCGTCACATCTCATCTACCTCCCGGTTTTAATGAATACG

ATTTTGTGCCAGAGTCCTTCGATAGGGACAAGACAATTGCACTGATCATGAACTCCTCTGGATCTACTGGTCTGCCTAAAGGTGTCGCTCTGCCTCATAGAACTGCCTGCGTGAGATTCTCGCATGCCAGAGATCCTATTTTTGGCAATCAAATCATTCCGGATACT

GCGATTTTAAGTGTTGTTCCATTCCATCACGGTTTTGGAATGTTTACTACACTCGGATATTTGATATGTGGATTTCGAGTCGTCTTAATGTATAGATTTGAAGAAGAGCTGTTTCTGAGGAGCCTTCAGGATTACAAGATTCAAAGTGCGCTGCTGGTGCCAACCCT

ATTCTCCTTCTTCGCCAAAAGCACTCTGATTGACAAATACGATTTATCTAATTTACACGAAATTGCTTCTGGTGGCGCTCCCCTCTCTAAGGAAGTCGGGGAAGCGGTTGCCAAGAGGTTCCATCTGCCAGGTATCAGGCAAGGATATGGGCTCACTGAGACTACA

TCAGCTATTCTGATTACACCCGAGGGGGATGATAAACCGGGCGCGGTCGGTAAAGTTGTTCCATTTTTTGAAGCGAAGGTTGTGGATCTGGATACCGGGAAAACGCTGGGCGTTAATCAAAGAGGCGAACTGTGTGTGAGAGGTCCTATGTGA**ACCGGT**AGGGC

CCTAGATCTTCTTGACGAGTTCTTCTGAATTATTAACGCTTACAATTTCCTGATGCGGTATTTTCTCCTTACGCATCTGTGCGGTATTTCACACCGCATCAGGTGGCACTTTTCGGGGAAATGTGCGCGGAACCCCTATTTGTTTATTTTTCTAAATACATTCAAATATGTAT

CCGCTCATGAGATTATCAAAAAGGATCTTCACCTAGATCCTTTTAAATTAAAAATGAAGTTTTAAATCAATCTAAAGTATATATGAGTAAACTTGGTCTGACAGTTACCAATGCGGTACCTCCCGGGA**AAGCTT**T<u>CGACATTTATAATGAACGTGAAT</u>TGCTCAACAGT

ATGGGCATTTCGCAGCCTACCGTGGTGTTCGTTTCCAAAAAGGGGTTGCAAAAAATTTTGAACGTGCAAAAAAAGCTCCCAATCATCCAAAAAATTATTATCATGGATTCTAAAACGGATTACCAGGGATTTCAGTCGATGTACACGTTCGTCACATCTCATCTAC

CTCCCGGTTTTAATGAATACGATTTTGTGCCAGAGTCCTTCGATAGGGACAAGACAATTGCACTGATCATGAACTCCTCTGGATCTACTGGTCTGCCTAAAGGTGTCGCTCTGCCTCATAGAACTGCCTGCGTGAGATTCTCGCATGCCAGAGATCCTATTTTTGGC

AATCAAATCATTCCGGATACTGCGATTTTAAGTGTTGTTCCATTCCATCACGGTTTTGGAATGTTTACTACACTCGGATATTTGATATGTGGATTTCGAGTCGTCTTAATGTATAGATTTGAAGAAGAGCTGTTTCTGAGGAGCCTTCAGGATTACAAGATTCAAAG

TGCGCTGCTGGTGCCAACCCTATTCTCCTTCTTCGCCAAAAGCACTCTGATTGACAAATACGATTTATCTAATTTACACGAAATTGCTTCTGGTGGCGCTCCCCTCTCTAAGGAAGTCGGGGAAGCGGTTGCCAAGAGGTTCCATCTGCCAGGTATCAGGCAAGGA

TATGGGCTCACTGAGACTACATCAGCTATTCTGATTACACCCGAGGGGGATGATAAACCGGGCGCGGTCGGTAAAGTTGTTCCATTTTTTGAAGCGAAGGTTGTGGATCTGGATACCGGGAAAACGCTGGGCGTTAATCAAAGAGGCGAACTGTGTGTGAGAGG

TCCTATGATTATGTCCGGTTATGTAAACAATCCGGAAGCGACCAACGCCTTGATTGACAAGGATGGATGGCTACATTCTGGAGACATAGCTTACTGGGACGAAGACGAACACTTCTTCATCGTTGACCGCCTGAAGTCTCTGATTAAGTACAAAGGCTATCAGGT

GGCTCCCGCTGAATTGGAATCCATCTTGCTCCAACACCCCAACATCTTCGACGCAGGTGTCGCAGGTCTTCCCGACGATGACGCCGGTGAACTTCCCGCCGCCGTTGTTGTTTTGGAGCACGGAAAGACGATGACGGAAAAAGAGATCGTGGATTACGTCGCCAG

TCAAGTAACAACCGCGAAAAAGTTGCGCGGAGGAGTTGTGTTTGTGGACGAAGTACCGAAAGGTCTTACCGGAAAACTCGACGCAAGAAAAATCAGAGAGATCCTCATAAAGGCCAAGAAGGGCGGAAAGATCGCCGTGTAATTCTAGAGTCGGGGCGGCCG

GCCGCTTCGAGCAGACATGATAAGATACATTGATGAGTTTGGACAAACCACAACTAGAATGCAGTGAAAAAAATGCTTTATTTGTGAAATTTGTGATGCTATTGCTTTATTTGTAACCATTATAAGCTGCAATAAACAAGTTAACAACAACAATTGCATTCATTTT

ATGTTTCAGGTTCAGGGGGAGGTGTGGGAGGTTTTTTAAAGC<u>AAGTAAAACCTCTACAAATGTGGTA</u>GCTAGCAT
